# The Relationship between Proinflammatory Molecules and PD-L1 in Patients with Obesity Who Underwent Gastric Sleeve Surgery—A Pilot Study

**DOI:** 10.3390/reports7030074

**Published:** 2024-09-03

**Authors:** Ciprian Cucoreanu, Ximena Maria Muresan, Adrian-Bogdan Tigu, Madalina Nistor, Radu-Cristian Moldovan, Ioana-Ecaterina Pralea, Maria Iacobescu, Cristina-Adela Iuga, Catalin Constantinescu, George-Calin Dindelegan, Constatin Ciuce

**Affiliations:** 1 Department of General Surgery, “Iuliu Hațieganu” University of Medicine and Pharmacy, 400012 Cluj-Napoca, Romania; 2 Department of Translational Medicine, Institute of Medical Research and Life Sciences—MEDFUTURE, “Iuliu Hațieganu” University of Medicine and Pharmacy, 400337 Cluj-Napoca, Romania; 3 Department of Proteomics and Metabolomics, Institute of Medical Research and Life Sciences—MEDFUTURE, “Iuliu Hațieganu” University of Medicine and Pharmacy, 400337 Cluj-Napoca, Romania; moldovan.radu@umfcluj.ro (R.-C.M.); iugac@umfcluj.ro (C.-A.I.); 4 Department of Pharmaceutical Analysis, Faculty of Pharmacy, “Iuliu Haţieganu” University of Medicine and Pharmacy, 400349 Cluj-Napoca, Romania; 5 Department of Anesthesia and Intensive Care, “Iuliu Haţieganu” University of Medicine and Pharmacy, 400012 Cluj-Napoca, Romania

**Keywords:** cytokines, PD-L1, obesity, bariatric surgery, inflammation, MCP-1

## Abstract

In the last few decades, obesity played a pivotal role by having a high impact on global economic and health systems due to its associated diseases, with cardiovascular, respiratory, musculoskeletal, oncological, mental, and social implications. One of the most incriminated physiopathological mechanisms in obesity is chronic inflammation. The primary goal of this pilot study was to determine the molecular aspects of inflammation among patients with obesity compared to participants with a normal BMI (≤25 kg/m^2^), as well as within a smaller subset of obese individuals who have been evaluated three months following sleeve gastrectomy. The research employs conventional blood tests and plasma measurements of particular molecules, such as proinflammatory cytokines and proteins that play critical roles in immune and inflammatory regulation. The results revealed a promising kinetic effect after bariatric surgery on IL-18, MCP-1, and PD-L1 molecules. The proinflammatory makers IL-18 (*p* = 0.006) and MCP-1 (*p* = 0.035) were elevated in the obese group compared to the control, while the follow-up group displayed lower levels of these molecules. Commonly investigated in oncology related studies, PD-L1 was recently linked to adipose tissue gain and its associated inflammatory effect. Until now, there is no clinical evidence for the relationship between circulating PD-L1 and proinflammatory markers derived from low-grade inflammation of the adipose tissue. The circulating PD-L1 levels were significantly lowered in the obese group compared to the control (*p* = 0.049), and after sleeve gastrectomy, the PD-L1 level increased. The present study is the first investigating this type of crosstalk and its potential involvement in bariatric patient management.

## 1. Introduction

According to the World Health Organization (WHO), in 2022, over 2.5 billion adults were overweight, including 890 million obese adults. These numbers represent 43% of the total adults, and the trend is not favorable compared to the situation in 1990, when only one out of four adults was overweight [1]. The escalating obesity epidemic is a serious public health challenge, and according to the World Obesity Atlas, by 2035, the global overweight and obesity prevalence will reach 51% [2].

Additionally, the current landscape highlights the importance of addressing the psychological and emotional aspects of obesity. Many individuals struggling with obesity may have underlying issues such as emotional eating, stress, or low self-esteem that contribute to their weight gain. Therefore, a comprehensive approach that includes behavioral therapy, counseling, and support groups is essential for long-term success in weight management. Furthermore, the rise of technology and digital health tools has provided new opportunities for individuals to track their progress, receive personalized recommendations, and connect with a community of like-minded individuals on their weight loss journey [3].

Medical factors, sleep factors, dietary factors, socioeconomic factors, and genetic factors are responsible for obesity. Different pathologies, such as hypothyroidism or Cushing syndrome, may cause weight gain and lower basal metabolism, leading to obesity [4,5]. Moreover, individuals may be affected by their lifestyle, characterized by a lack of physical exercise, insufficient sleep, or high caloric intake that could trigger metabolic dysfunctions and promote weight gain leading to obesity [6,7].

As a multifactorial disease, obesity may also be related to genetic factors. Studies show that single nucleotide polymorphisms in fat mass and obesity-associated (FTO) genes were found to be involved in diabetes development [8]. Moreover, other genes may be associated with weight gain, while others involved in epigenetic mechanisms and inflammation may significantly contribute to obesity development. Epigenetic alteration related to the methylation status of the DNA, can modulate and trigger biological processes such as adipogenesis and inflammatory processes [9].

The link between increased nutrient intake and activation of the innate immune system has been intensely debated, and it has been demonstrated that it could be a key mechanism in energy imbalance [10]. The lipid storage organ, white adipose tissue (WAT), is crucial in obesity-associated disorders due to the potential lipotoxicity induced in non-specialized organs [11]. When the adipose tissue gains volume, the proinflammatory terrain is triggered by the infiltrated macrophages, which produce proinflammatory cytokines and impair insulin signaling and may trigger other mechanisms that maintain the inflammation loop within the adipose tissue [12].

Proinflammatory cytokines stimulate inflammation and maintain the proinflammatory terrain in adipose tissue via signaling through tumor necrosis factor alpha (TNFα), interferon (IFN), oncostatin-M (OSM), and interleukins (IL-1β, IL-6, IL-8, IL-18, IL-33) [13,14]. Aside from cytokine signaling, other molecular pathways are involved in obesity development and trigger proinflammatory mechanisms in an indirect manner. Monocyte chemoattractant protein-1 (MCP-1) is usually elevated in patients with obesity and is correlated with the elevated macrophage infiltration within fat tissue; thus, the high levels of MCP-1 are associated with increased inflammatory status [15,16]. Regulated on activation, normal T cells expressed and secreted (RANTES) are another inflammatory molecule that may be related to obesity and chronic inflammation due to its role in multiple inflammatory disorders like atherosclerosis, atopic dermatitis, asthma, or endometriosis, linking inflammation signaling with the immune system [17,18].

As the overweight population has an increased risk of developing malignancies due to several dysfunctions such as hypoxia, prolonged inflammation, and metabolic imbalance, the study of programmed death ligand 1 (PD-L1) in obesity could represent an important step for further patient management [19,20,21,22]. In addition, some studies suggest that PD-L1 may have a role in reducing adipose tissue inflammation and limiting diet-induced obesity [21]. The adipose tissue macrophage infiltration and activation process is a hallmark of derived and induced local inflammation and is linked with adipocyte death [23], thus PD-L1 may play an important role in obesity-related alterations of the inflammatory mechanisms.

PD-L1, a key immune checkpoint protein, plays a critical role in modulating immune responses by inhibiting T-cell activity. In obesity, the downregulation of PD-L1 observed in adipose tissue and immune cells reflects an altered immune environment characterized by chronic low-grade inflammation. This reduced expression of PD-L1 in obesity may compromise immune tolerance and exacerbate inflammation, contributing to the metabolic dysregulation associated with obesity [22,24].

The downregulation of PD-L1 is influenced by the inflammatory cytokines prevalent in obesity, such as TNF-α and IL-6, which can directly impact PD-L1 expression and function. Additionally, the shift towards a proinflammatory macrophage phenotype within adipose tissue further supports the reduced PD-L1 expression. Understanding these mechanisms highlights the complex interplay between immune regulation and metabolic disorders and suggests that targeting PD-L1 pathways could be a potential strategy for managing obesity-related inflammation and its associated health complications [22,25].

Obese patient management is crucial and needs continuous improvements to guarantee better outcomes after bariatric surgery and noninvasive anti-obesity therapies. Thus, we aim to provide insights into the molecular aspects of obesity and how bariatric surgery may improve the proinflammatory terrain.

Bariatric surgery (BS) serves as an important strategy for facilitating weight loss in individuals with obesity. The metabolic changes that ensue after the surgery play a significant role, as they broaden the scope of benefits beyond just weight loss. Reflecting its nomenclature, “metabolic surgery” also aims to address metabolic disorders associated with obesity [26]. Evidence suggests that bariatric surgery is more effective in achieving substantial weight loss and in managing overall metabolic irregularities than in the standard of care (lifestyle changes, specific weight loss medication, exercise). It is noteworthy that the effects persist for years following the surgical procedure. Furthermore, there is considerable evidence that supports the role of bariatric surgery in achieving the remission of diabetes in patients suffering from type 2 diabetes mellitus (T2DM). The primary rationale for bariatric surgery is to facilitate weight reduction in individuals who have been unsuccessful in achieving weight loss through non-surgical interventions. Consensus guidelines specify that bariatric surgery is suitable for patients with a body mass index (BMI) exceeding 40 kg/m^2^, as well as for those with a BMI greater than 35 kg/m^2^ who present with related comorbid conditions. Among the various bariatric surgical options, the Roux-en-Y Gastric Bypass (RYGB) and sleeve gastrectomy (SG) are the most frequently performed. RYGB involves the surgical division of the stomach to establish a gastric pouch that holds about one ounce and a redistribution of the small intestine in order to shorten the passage length through the digestive tube necessary for the absorption of nutrients (restrictive and malabsorptive weight loss mechanism). Alternatively, SG is characterized by the excision of nearly 80% of the stomach, leading to the formation of a tubular stomach (restrictive weight loss mechanism) [27,28,29].

The objective of this pilot study is to perform a molecular-level analysis of inflammation in patients with obesity compared to participants without obesity (controls), as well as in a smaller group of obese individuals evaluated three months after undergoing sleeve gastrectomy. The investigation utilizes standard blood tests and plasma quantification of specific molecules, including proinflammatory cytokines and proteins essential to immune and inflammatory regulation.

## 2. Material and Methods

### 2.1. Patient Selection and Follow-Up

Study design: Following ethics board approval (No. AV73/08.11.2021), a prospective observational design was adopted. The study was conducted at the Clinical County Emergency Hospital Cluj, Romania, between January 2024 and June 2024. The admission for elective surgery included patient screening for eligibility. Patients included as obese were selected for bariatric surgery. The inclusion criteria included consent to participate, age above 18 (adult persons), body mass index (BMI) ≥ 35 kg/m^2^, and being proposed for gastric sleeve surgery (laparoscopic surgery). Non-patients with obesity, referred to as controls, were healthy adults (BMI ≤ 25 kg/m^2^) admitted during the same period for routine periodical medical evaluation [30]. The participants within the obese group had comorbidities associated with obesity (type 2 diabetes, hypertension, dyslipidemia, sleep apnea). The control group had no comorbidities at all. As exclusion criteria for the selected patients, the following medical history was considered: severe chronic inflammatory diseases, autoimmune disease, iron supplementation, oncological disease, endocrine diseases, or gastrointestinal tract bleeding. The control group had an almost even number of males and females with a similar age range and hormonal status compared to the obese group.

Follow-up: the patients that underwent sleeve gastrectomy were evaluated after three months post-surgery. The parameters measured during follow-up included all preoperative analyses and the molecular determination for proinflammatory molecules and PD-L1.

Study sample: baseline characteristics such as age, sex, BMI, and comorbidities were determined, together with hospitalization stay. Upon admission, routine blood samples were taken and used for the following lab tests: liver function, full blood count, urea, uric acid, creatinine, electrolytes, total proteins, lipid profile, albumin, and iron. All blood parameters were determined in the hospital’s laboratory using a Mindray BC6200 Hematology analyzer (Mindray, Shenzhen, Mainland China) and Beckman Coulter AU680 analyzer (Beckman Coulter, Brea, CA, USA) following standard protocols, and the results are presented in the Section 3.

### 2.2. Sample Collection, Processing, and Storage Molecular Analysis

The peripheral blood was collected in heparin-coated vacutainers (BD, Franklin Lakes, NJ, USA—cat no. 367876, batch 3195160), and the plasma was collected by centrifugation at 3000× *g* for 15 min at 4 °C within the first two hours after blood collection. The plasma was aliquoted in 1.5 mL sterile tubes and stored frozen at −80 °C until further use.

The possibility of the endotoxin contamination of heparin has been reported before [31] and was considered when designing the sample collection and processing. All samples were processed within a maximum of 2 h from collection, therefore limiting the risk of cytokine release due to endotoxin contamination.

### 2.3. Quantification of Circulating Proinflammatory Cytokines with Immunoenzymatic Testing (ELISA)

Plasma samples were tested according to the manufacturers protocols for the following proinflammatory markers: IL-8 (RD194558200R) (Biovendor, Brno, Czech Republic), IL-18 (Cat. no. RAF143R) (Biovendor, Brno, Czech Republic), MCP-1 (SEH00192A, Ref. no. 336151) (Qiagen, Hilden, Germany), RANTES (SEH00703A, Ref. no. 336151) (Qiagen, Hilden, Germany), PD-L1/B7-H1 (Cat. no. DB7H10) (R&D Systems, Minneapolis, MN, USA). The plates were measured at 450 nm using a TECAN SPARK 10M spectrophotometer (Männedorf, Switzerland). The final concentrations for each sample were calculated considering the dilution factor applied for each individual assay.

### 2.4. Statistical Analysis

Statistical analyses were performed using Rstudio (Posit Software, version 2024.04.1+748). The different patient characteristics were compared between obese and non-obese (control) using the Welch two sample *t*-test (comparing the two independent groups which are not assumed to be equal with continuous data), the Wilcoxon rank sum exact test, and the Wilcoxon rank sum test (for comparison between independent groups with a non-normal distribution). Descriptive statistics were obtained. Values were expressed as medians and IQRs. A heatmap was performed using the ggplot2 function, where darker red indicates larger positive differences and darker blue indicates larger negative differences, with larger differences being more impactful. Multiple regression analysis for each variable was performed using the library (MASS) function, concerning predictor variables such as control cohort, BMI, age, and sex. To compare baseline obesity and follow-up groups (the same patient ID), we have used two types of *p*-values, one from a paired *t*-test and one from a Wilcoxon signed-rank test. For statistical significance, a cutoff value of *p* < 0.05 was selected.

## 3. Results

### 3.1. Clinical Parameters and Molecular Analysis

**Figure 1:**
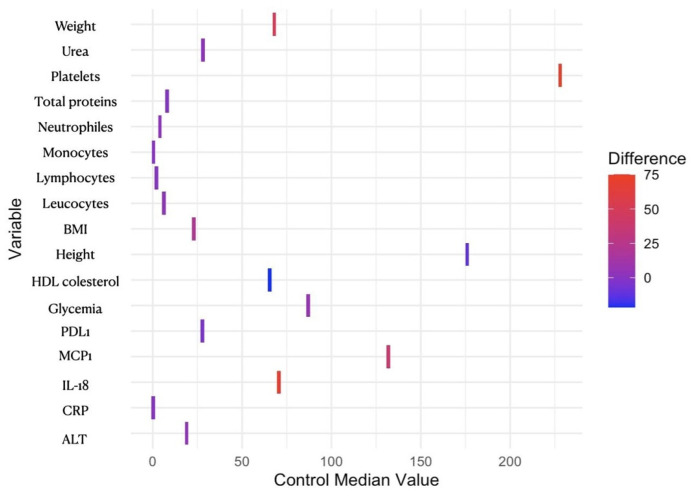
Heatmap of median differences between obese and control cohorts. The color gradient from blue to red represents the difference between the median values of the obese group and the control group for each variable. The median value was calculated for the obese group using the same method as for the control group, and further subtracted the control group’s median from the obese group’s median for each variable. Blue indicates a smaller or negative difference, while red indicates a larger or positive difference. Variables with tiles that are more towards red have a larger positive difference (the obese group has higher values), while variables with tiles that are more towards blue have a smaller or negative difference (the obese group has lower values). HDL—high density lipoprotein; BMI—body mass index; PDL1—programmed death-ligand 1; MCP1—monocyte chemoattractant protein-1; IL-18—interleukin 18; CRP—C-reactive protein; ALT—alanine aminotransferase/alanine transaminase.

A group of 54 patients were included in our study (31 patients with obesity and 23 participants without obesity), and the baseline characteristics are presented in Table 1. The clinical and molecular characteristics of patients with obesity and controls, presented in Table 1 and Figure 1, present the routine analysis results and the molecular analysis of the proinflammatory molecules and PD-L1 by comparing the median values for each group. For the follow-up after bariatric surgery (gastric sleeve), 16 patients were reevaluated after three months.

The proinflammatory status in the obesity group is highlighted by the elevated circulating proinflammatory molecules that were quantified by ELISA, with IL-18 and MCP-1 as the only statistically significant changes between groups (obese vs. control). Circulating PD-L1 levels seem lower in the obese group compared to the controls, while significant increases in proinflammatory markers such as CRP (*p* < 0.001), IL-18 (*p* = 0.006), and MCP-1 (*p* = 0.035) were observed.

IL-8 and RANTES displayed no differences between groups. Clinical parameters such as monocyte, leukocyte, neutrophil, and platelet counts were significantly different.

Total proteins and HDL were significantly increased in controls compared to the obese group. On the other hand, glycemia was higher in the obese group. All analyses are presented in Table 1 and further discussed.

The median differences between obesity and the control group for parameters that are significantly modified are presented in Figure 1. Platelet counts, MCP-1, and IL-18 highlight large positive differences between the groups, as these parameters were significantly increased in patients with obesity.

### 3.2. Multivariate Analysis

In order to understand if there is a relationship between variables, we undertook a regression analysis in which we compared the available variables with the control group (the difference between the control and obese), age (the effect of age on the variable), sex (the difference between males and females), and BMI (the effect of BMI on the respective variable). The statistical analysis is available in the Supplementary File.

Age is a significant predictor for MCP-1 and glycemic levels. The intercept is significant for IL-18 and PD-L1 levels. The control cohort is significant for IL-18. The predictors demonstrate limited significance on albumin, although sex appears to be nearly significant. The predictors are more influential on total proteins, especially the cohort and sex, indicating a significant effect of these factors. Creatinine and HDL cholesterol models show the strongest performance, with significant predictors and higher R-squared values. The ALAT/TGP, ASAT/TGO, and triglyceride models have lower R-squared values, indicating they explain less variability in their respective response variables. LDL cholesterol and total cholesterol models have mixed results, with some significant predictors but relatively lower R-squared values. The control cohort (control vs. obese) is significantly associated with lower levels of leukocytes and lymphocytes and marginally with lower monocyte levels. The model explains a significant portion of the variance in some response variables (e.g., leukocytes, neutrophils), as indicated by the R-squared values, but not in others (e.g., lymphocytes, monocytes).

**Figure 2:**
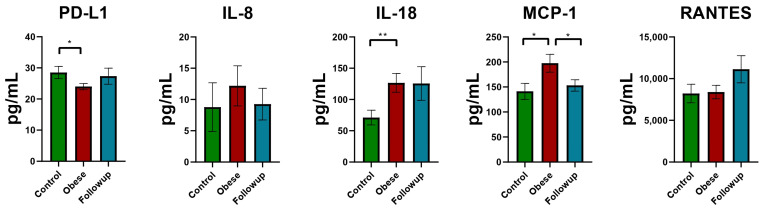
Proinflammatory molecules and PD-L1 determinations—comparison between the three groups. Statistical evaluation was performed using GraphPad Prism (version 8) applying an unpaired two-tailed *t*-test with Welch’s correction, and the results are expressed as mean value ± SEM, * *p* < 0.05; ** *p* < 0.01.

**Figure 3:**
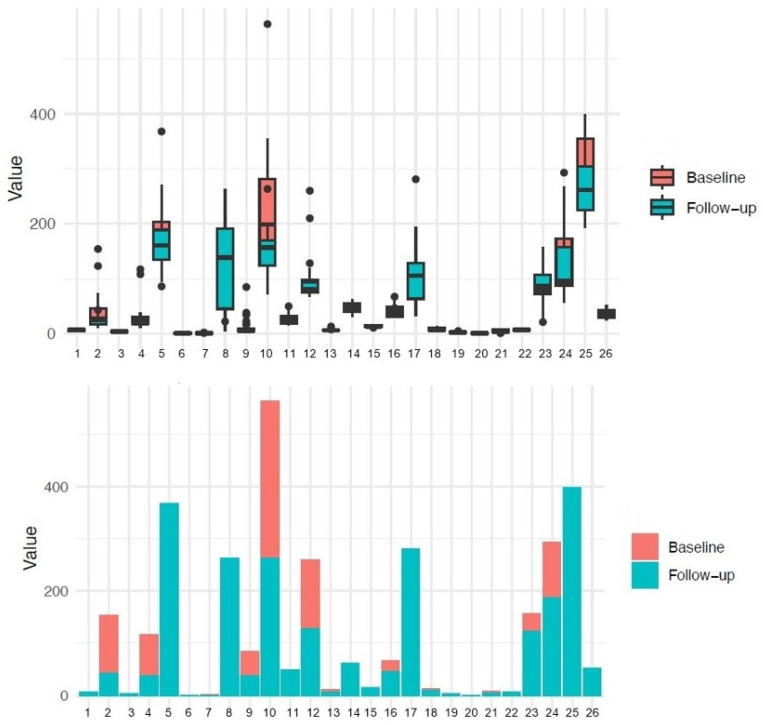
Comparison of variables at baseline and follow-up, including all measured parameters. The upper panel displays box plots for each variable listed in the legend, which represents the interquartile range (IQR), which contains the middle 50% of the data. The line inside the box represents the median value. The lower panel shows bar plots for each variable. They represent the central tendency or the mean/median value for the baseline and follow-up measurements. Legend: 1. uric acid (mg/dL); 2. ALT (U/L); 3. albumin (g/dL); 4. AST (U/L); 5. total cholesterol (mg/dL); 6. creatinine (mg/dL); 7. CRP (mg/dL); 8. IL-18 (pg/mL); 9. IL-8 (pg/mL); 10. MCP-1 (pg/mL); 11. PD-L1 (pg/mL); 12. glucose (mg/dL); 13. HbA1c (%); 14. HDL (mg/dL); 15. hemoglobin (g/dL); 16. BMI (kg/m^2^); 17. LDL (mg/dL); 18. leukocytes (10^9^/L); 19. lymphocytes (10^9^/L); 20. monocytes (10^9^/L); 21. neutrophils (10^9^/L); 22. total proteins (g/dL); 23. iron (ug/dL); 24. triglycerides (mg/dL); 25. thrombocytes (10^9^/L); 26. urea (mg/dL).

The results of baseline/follow-up analysis using paired *t*-test and Wilcoxon test are presented in Figure 2 and Figure 3. The following variables were significant: MCP-1, triglycerides, CRP, HDL cholesterol, hemoglobin, BMI, leukocytes, monocytes, and neutrophils. In contrast, variables with no statistical significance include ALAT/TGP, ASAT/TGO, uric acid, albumin, total cholesterol, creatinine, IL-18, IL-8, PD-L1, glycemia, LDL cholesterol, lymphocytes, total proteins, platelets, and urea.

Regarding the hospital stay parameter, all the correlation coefficients are very weak (close to 0), indicating no linear relationship between days of hospitalization and the concentrations of the measured molecules (IL-18, IL-8, MCP-1, PD-L1, RANTES).

**Figure 4:**
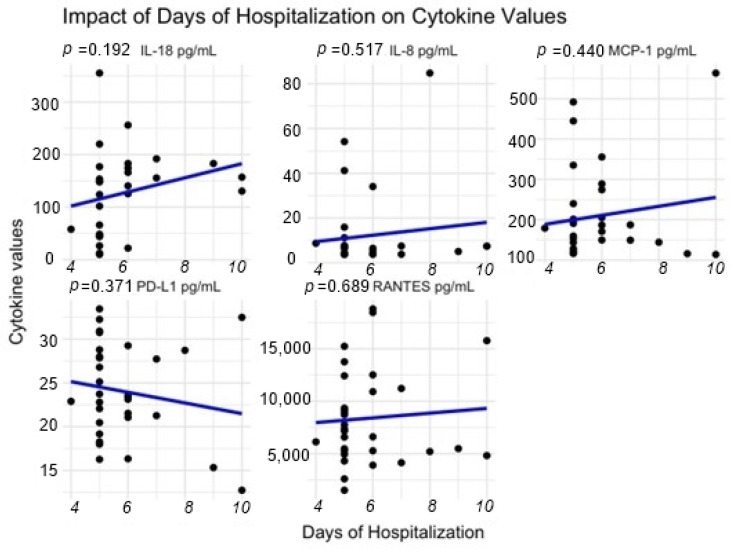
Correlation between hospital stay and PD-L1 and proinflammatory molecules.

As depicted in Figure 4, the values for IL-8, MCP-1, and RANTES showed a very weak positive relationship without a statistically significant *p* value, while PD-L1 reveals a weak negative relationship correlation.

## 4. Discussion

The main findings of the present study highlight the well-known proinflammatory status related to obesity and validated in our studied group through high levels of IL-18, MCP-1, CRP, monocytes, lymphocytes, and neutrophils compared with the control group. A kinetic decrease was observed three months after sleeve gastrectomy in terms of IL-18 and MCP-1 molecules. The follow-up for the circulating PD-L1, which was decreased preoperatively, shows increased values comparable with the control, even though it is not statistically significant. Regarding the hospital length of stay, we observed that the concentrations of IL-18, IL-8, MCP-1, and RANTES increase with the duration of hospitalization, while PD-L1 has the opposite effect, but again, without statistical significance.

As a potential mechanism, the innate and adaptive immune system cells are affected by the proinflammatory terrain in obesity, with some cells being recruited in the adipose tissue and participating in chronic inflammation development (infiltrating macrophages, neutrophils, and lymphocytes), thus stimulating the production of proinflammatory molecules that may trigger downstream signaling pathways responsible for the immune defense. Due to this imbalance in the immunologic pathways, PD-L1 expression could be decreased, and its circulating levels will be lower than in non-obese subjects. After weight loss, this proinflammatory terrain will be decreased, and therefore the immune mechanism will be restored. All in all, further investigations are needed to confirm these observations and validate PD-L1 as a potential biomarker for obesity management and its cross-talk with proinflammatory markers.

The global rise in obesity prevalence points out the urgent need for improved treatment strategies. Developing novel strategies to enhance patient care and mitigate obesity-related issues requires multidisciplinary action and personalized therapies. The relationship between obesity and chronic inflammation gained attention during the last decades, being demonstrated that inflammatory terrain maintains a loop of high-risk increased proinflammatory signaling in the adipose tissue, triggering the positive feedback that sustains obesity and its derived disorders. In contrast to acute inflammation, which is characterized by swelling and white blood cell movement, chronic inflammation lasts longer and is identified by the infiltration of lymphocytes and macrophages, two important adipose tissue constituents [32].

The molecular mechanisms of adipocyte inflammation imply several signaling pathways that may interfere with apoptosis, cell cycle, oxidative stress, or pathways that may trigger oncogenes [33,34]. Furthermore, adipose tissue that harbors infiltrated immune cells can trigger the cytokine and chemokine release by activating macrophages and neutrophils. Then, the adaptive immune system cells, B and T cells, will be activated by specific signals, enhancing the inflammatory state in the adipose tissue due to the low levels of anti-inflammatory cytokines and increased levels of proinflammatory ones [35].

Obesity is defined as excessive lipid storage, which will cause severe stress within the adipocytes. The cellular increase in size is well tolerated until a certain level, beyond which adipocytes develop molecular dysfunction and death. Adipocytes in visceral adipose tissue become dysfunctional at a smaller size than subcutaneous adipocytes [36]. One important molecular malfunction observed in stressed adipocytes is their response to hypoxia [37,38]. In severe weight gain, adipose tissue is characterized by low levels of oxygen, thus resulting in hypoxia activation, triggering the proinflammatory response. Therefore, stressed adipocytes release large amounts of proinflammatory adipokines and cytokines, such as leptin, IL-6, tumor necrosis factor alpha (TNF-α), IL-1, IL-8, and monocyte chemoattractant protein 1 (MCP-1), which further promote inflammation. The proinflammatory terrain induces low-grade inflammation in obese adipose tissue, which will be further amplified by the immune system cells [39].

Sinclair et al. described the metabolic effects of bariatric surgery and highlighted the benefits of surgery in patients suffering from obesity. The most striking effect after sleeve gastrectomy is weight loss and fat mass loss. The two main adipokines involved in the metabolic effect of surgery are leptin and adiponectin. Adiponectin levels increase after surgery and leptin levels decrease, being correlated with weight loss and caloric restrictions. Moreover, after sleeve gastrectomy, several changes were observed in different analytes: the increased level of adiponectin was correlated with lowered IL-6 and CRP; furthermore, other parameters such as insulin secretion and HDL were increased, while total cholesterol, LDL, triglycerides, glucagon, and AST/ALT had lower concentrations. All in all, it may indicate that glucose and fatty acid metabolism could be restored after bariatric surgery, and the inflammation signaling indicates that the inflammatory status is improving [40]. In obesity, changes in adipokine balance can be translated into an overactivated immune system that could be associated with metabolic imbalance, while under normal conditions and weak inflammatory terrain, the adipose tissue can maintain the adipokine balance and prevent the immune cells hyperactivation [41]. The imbalance in leptin and adiponectin leads to increased nutrient uptake, and it is finally translated into increased inflammatory terrain in adipose tissue, which is characterized by infiltrated immune cells that produce proinflammatory molecules and maintain the metabolic dysfunction loop. Bariatric surgery can restore metabolic balance by reducing the body fat and lowering the inflammatory terrain in the adipose tissue. The reduced inflammation has a direct effect on the immune cells, which will create a favorable metabolic and immunologic context to restore normal physiological conditions after surgery, and with controlled nutrient intake, the body fat can be under control [42,43].

In the context of known and investigated adipocyte inflammatory pathways and signaling, referring to IL-1, IL-6, TNF-α, IL-10, leptin, and adiponectin [13,44,45] our aim was to look over other involved molecules as IL-8, IL-18, MCP1, RANTES, and PD-L1, with potential clinical utility [46]. Data revealed by Li et al. [47] talk about adipocyte promotion of tumor progression via PD-L1 expression due to TNF-α/IL-6 signaling. In this context of immune modulation, regulated upon activation, normal T cells expressed and secreted (RANTES) may present alteration during the accelerated process of losing weight after bariatric surgery. Monocyte chemoattractant proteins, MCP1, have been repeatedly implicated in atherosclerotic lesion development [48]. Modification of MCP1 levels following bariatric surgery may possibly suggest a future pathway to assess the cardiovascular risk before and after bariatric surgery [49].

Interleukin 18 belongs to the IL-1 family of cytokines, a group that promotes the activity of the innate immune system. IL-18 stimulates both the innate and acquired immune responses. IL-8 is a chemoattractant cytokine produced by a variety of tissue and blood cells. Unlike many other cytokines, it has a distinct target specificity for the neutrophil. As neutrophil counts were different between groups and the NLR was stated in many studies to be a good indicator of chronic obesity-related inflammation [50] and the intricate and directly proportional elevated risk for developing postoperative complications [51,52], we proposed to analyze the circulating levels in the extent of possible future use of its predictive values. We observed increased levels of circulating IL-18 in our obese group compared to the controls, confirming that the proinflammatory status was active in patients with obesity. In the case of IL-8, the circulating levels were increased without being statistically significant. Circulating levels of MCP-1 were significantly higher in the obese group compared to the controls, suggesting that signals for monocyte recruitment were increased, this being correlated with the proinflammatory status of the obese group. Both IL-18 and MCP-1 are correlated with increased CRP, monocyte count, total platelet count, and total triglycerides, as the results are presented in Table 1.

The evaluation of PD-L1 as a potential biomarker for estimating the inflammation grade in patients with obesity, revealed that the circulating levels of PD-L1 were lower in the obese group, in contrast with the elevated levels of proinflammatory markers.

The proinflammatory status of the patients with obese participants was compared at baseline and at follow-up, only for those that had follow-up determinations. Thus, we observed that the circulating levels of IL-18, IL-8, and MCP-1 followed a downward trend, with MCP-1 being the only molecule that displayed significant changes. In terms of PD-L1, we observed an increased level of circulating molecules, results that may indicate its potential regulatory effect on proinflammatory molecules such as cytokines from the IL-1 family (IL-6, IL-8, IL-18) or TNFα.

Taken together, the results obtained for baseline compared to follow-up revealed that PD-L1 had higher levels in the follow-up group, related to the decreased concentration of IL-8, MCP-1, and IL-18. Furthermore, after three months postoperatively, the BMI was significantly lower, and CRP, triglycerides, total leukocytes, lymphocytes, monocytes, and neutrophils were decreased compared to baseline, while HDL and PD-L1 displayed slightly increased values.

The molecular findings, together with routine analysis performed at baseline and follow-up indicate that gastric sleeve operation positively contributes to patients outcome, playing a role in decreasing the proinflammatory status in an early postoperative stage (after three months). The high preoperative MCP-1 levels compared to the controls and the observed lower level of MCP-1 in the follow-up group can be explained by a modulation to a decreased level of circulating leukocytes leading to an improvement in the general inflammatory status of the patients with obesity after surgery. The discussion may be developed in the direction of a potential use of these associations between MCP-1 and leukocytes, together with circulating PD-L1, in order to assess clinical variables in bariatric patients. In what concerns hospitalization stay, with the limitation of a very small follow-up group, we observed a weak correlation with no linear relationship with proinflammatory molecules and PD-L1. However, the prolonged hospitalization marks a higher level of MCP-1, IL-18, and IL-8 and low levels of PD-L1. This may be of clinical use as a potential predictor only after validation on large cohorts of bariatric patients and different time-points of follow-up.

The results referring to the improvement of the glycemic and lipid profiles were consistent with the literature data [52,53,54,55], with a significant difference between the control and obese groups and a notable improvement trend within the follow-up group. In terms of total proteins and albumin levels, our results displayed no variation between baseline and follow-up, even though the literature links hypoalbuminemia and hypoproteinemia with obesity and a higher risk of developing postoperative complications in cases of low albumin levels [56,57].

Regarding the limitation of the current study, firstly, although we observed some trends in our analysis, the small sample of the groups could be responsible for the lack of statistical significance, which requires further research with a higher number of subjects to validate our findings. Other proinflammatory markers should be assessed (IL-1, IL-6, TNFα, or Interferon) to completely characterize the picture of inflammation in patients with obesity. Secondly, longer periods of follow-up should be included in the design of future studies for a better overview of the benefits of bariatric surgery and the possibility to use such molecules as prognostic tools. Furthermore, a multicentric study with different geographical and ethnical areas due to different heterogenicity among obese individuals could improve knowledge in this area of research.

## 5. Conclusions

Until now, there is no scientific evidence for an investigation model questioning the relationship between circulating proinflammatory markers, PD-L1, and gastric sleeve procedure. In this pilot study, we confirmed that the proinflammatory markers are increased in patients with obesity, and following surgery, their concentrations decrease, with a tendency towards normalization. On the other hand, in terms of PD-L1, the trend was to increase after sleeve gastrectomy, a fact that highlights an improvement in lowering general inflammation and could be of interest as a biomarker for further studies.

## Figures and Tables

**Table 1:** Clinical and molecular characteristics of patients with obesity and controls.

Patient Characteristics by Cohort			
Obese vs. Control Patients			
Patient Characteristics	Obese, N = 31 ^1^	Control, N = 23 ^1^	*p*-Value
Age	42 (30, 54)	36 (28, 50)	0.300
Sex			0.052
Females	24 (77%)	12 (52%)	
Males	7 (23%)	11 (48%)	
Weight (kg)	117 (106, 140)	68 (62, 74)	<0.001 ***
Height (cm)	164 (160, 172)	176 (164, 180)	0.011 *
BMI (kg/m^2^)	45 (40, 47)	23 (21, 24)	<0.001 ***
IL-8 (pg/mL)	7 (4, 8)	4 (4, 5)	0.500
IL-18 (pg/mL)	140 (48, 174)	70 (25, 102)	0.006 **
RANTES (pg/mL)	7175 (5244, 11,070)	7231 (5939, 9166)	>0.900
PD-L1 (pg/mL)	23 (21, 28)	28 (22, 35)	0.049 *
MCP-1 (pg/mL)	171 (127, 222)	132 (99, 191)	0.035 *
Hemoglobin (g/dL)	13.90 (13.30, 14.65)	13.90 (12.65, 15.10)	0.900
Leukocytes (10^9^/L)	9.59 (8.23, 10.72)	6.19 (5.44, 6.65)	<0.001 ***
Neutrophils (10^9^/L)	6.27 (5.15, 7.12)	4.04 (3.54, 4.65)	<0.001 ***
Lymphocytes (10^9^/L)	2.52 (2.19, 3.05)	1.99 (1.74, 2.29)	0.001 ***
Monocytes (10^9^/L)	0.59 (0.44, 0.65)	0.38 (0.33, 0.43)	<0.001 ***
Platelets (10^9^/L)	303 (254, 346)	228 (193, 246)	<0.001 ***
CRP (mg/dL)	0.73 (0.58, 0.95)	0.26 (0.13, 0.38)	<0.001 ***
Albumin (g/dL)	4.11 (3.90, 4.28)	4.56 (4.38, 4.63)	0.300
Total protein (g/dL)	6.94 (6.73, 7.41)	7.94 (7.92, 7.97)	0.022 *
Glucose (mg/dL)	94 (82, 104)	87 (78, 96)	0.022 *
ALT (U/L)	23 (18, 36)	19 (15, 30)	0.031 *
AST (U/L)	22 (18, 28)	21 (16, 25)	0.200
Urea (mg/dL)	31 (26, 39)	28 (25, 33)	0.046 *
Creatinine (mg/dL)	0.69 (0.61, 0.83)	0.81 (0.76, 0.95)	0.063
Uric acid (mg/dL)	5.93 (5.05, 6.47)	3.75 (3.75, 3.75)	0.150
HDL cholesterol (mg/dL)	44 (40, 52)	66 (57, 72)	<0.001 ***
LDL cholesterol (mg/dL)	105 (78, 122)	112 (94, 123)	0.300
Total cholesterol (mg/dL)	183 (141, 205)	180 (164, 210)	0.300
Triglycerides (mg/dL)	131 (103, 179)	86 (62, 120)	0.068
Iron (ug/dL)	79 (61, 100)	77 (62, 86)	>0.900

^1^ Median (IQR); n (%); significant differences regarding groups * *p* < 0.05, ** *p* < 0.01, *** *p* < 0.001.

## Data Availability

The original data presented in the study are included in the article, further inquiries can be directed to the corresponding authors.

## Supplementary Materials

The following supporting information can be downloaded at: https://www.mdpi.com/article/10.3390/reports7030074/s1, Supplementary File—Summary of Multivariate Linear Regression Analysis.
