# Supplementary material for: The Relationship between Proinflammatory Molecules and PD-L1 in Patients with Obesity Who Underwent Gastric Sleeve Surgery—A Pilot Study
Source: Reports (MDPI). 2024 Sep 3;7(3):74. doi: 10.3390/reports7030074 (PMC12225238; doi:10.3390/reports7030074)
Supplement: Supplementary file 1 [file reports-07-00074-s001.zip › reports-3197562-supplementary.pdf]

Summary of Multivariate Linear Regression Analysis

To interpret the output from the regression analysis (tidy_glicemie), you should understand the meaning of each term in the table:

Terms:

- **(Intercept)**: This is the baseline level of the response variable when all predictors are at zero.
- **Cohortcontrol**: This term represents the difference in Glicemie between the control cohort and the reference cohort.
- **Varsta**: This term shows the effect of age (Varsta) on the variable.
- **SexM**: This term indicates the difference in the variable between males (SexM) and the reference sex (likely females if coded that way).
- **book$IMC (kk/m2)**: This term represents the effect of BMI (IMC) on the variable.

Standard Errors:

- The **std.error** column provides the standard errors of the coefficients, which measure the average distance that the observed values fall from the regression line.

t-values (Statistic):

- The **statistic** column shows the t-statistic for each coefficient, calculated as the estimate divided by the standard error. This indicates how many standard deviations the coefficient is away from zero.

p-values:

- The **p.value** column indicates the probability of observing a test statistic as extreme as, or more extreme than, the observed value under the null hypothesis that the coefficient is zero. This helps determine the statistical significance of each coefficient.

Specific Interpretation for Each Response Variable

Response book$Leucocite (10^9/L)``

- **Cohortcontrol**:
  - Estimate: -2.82253
  - P-value: 0.033181 (significant at the 0.05 level)
  - Interpretation: Being in the control cohort is associated with a decrease in Leucocite levels by approximately 2.82253 units, compared to the obese cohort.
- **Varsta (Age)**:
  - Estimate: -0.02811
  - P-value: 0.167283 (not significant)
  - Interpretation: Age does not have a statistically significant effect on Leucocite levels.
- **SexM (Male)**:
  - Estimate: 0.22977
  - P-value: 0.710179 (not significant)
  - Interpretation: Sex does not have a statistically significant effect on Leucocite levels.
- **IMC (BMI)**:
  - Estimate: 0.03096
  - P-value: 0.536230 (not significant)
  - Interpretation: BMI does not have a statistically significant effect on Leucocite levels.

Response book$Neutrofile (10^9/L)``

- **Cohortcontrol**:
  - Estimate: -1.09164
  - P-value: 0.2969 (not significant)
  - Interpretation: Being in the control cohort is not statistically significantly associated with Neutrofile levels.
- **Varsta (Age)**:
  - Estimate: -0.01874
  - P-value: 0.2507 (not significant)
  - Interpretation: Age does not have a statistically significant effect on Neutrofile levels.
- **SexM (Male)**:
  - Estimate: 0.42605
  - P-value: 0.3928 (not significant)
  - Interpretation: Sex does not have a statistically significant effect on Neutrofile levels.
- **IMC (BMI)**:
  - Estimate: 0.05561
  - P-value: 0.1704 (not significant)
  - Interpretation: BMI does not have a statistically significant effect on Neutrofile levels.

Response book$Limfocite (10^9/L)``

- **Cohortcontrol**:
  - Estimate: -1.078279
  - P-value: 0.0212 (significant at the 0.05 level)
  - Interpretation: Being in the control cohort is associated with a decrease in Limfocite levels by approximately 1.078279 units, compared to the obese cohort.
- **Varsta (Age)**:
  - Estimate: -0.007845
  - P-value: 0.2713 (not significant)
  - Interpretation: Age does not have a statistically significant effect on Limfocite levels.
- **SexM (Male)**:
  - Estimate: 0.023176
  - P-value: 0.9150 (not significant)
  - Interpretation: Sex does not have a statistically significant effect on Limfocite levels.
- **IMC (BMI)**:
  - Estimate: -0.019592
  - P-value: 0.2677 (not significant)
  - Interpretation: BMI does not have a statistically significant effect on Limfocite levels.

Response book$Monocite (10^9/L)``

- **Cohortcontrol**:
  - Estimate: -0.1940335
  - P-value: 0.06865 (marginally significant at the 0.1 level)
  - Interpretation: Being in the control cohort is associated with a decrease in Monocite levels by approximately 0.1940335 units, compared to the obese cohort.
- **Varsta (Age)**:
  - Estimate: -0.0001482
  - P-value: 0.92760 (not significant)
  - Interpretation: Age does not have a statistically significant effect on Monocite levels.
- **SexM (Male)**:
  - Estimate: 0.0557120
  - P-value: 0.26825 (not significant)
  - Interpretation: Sex does not have a statistically significant effect on Monocite levels.
- **IMC (BMI)**:
  - Estimate: 0.0008444
  - P-value: 0.83462 (not significant)

- Interpretation: BMI does not have a statistically significant effect on Monocite levels.

ELISA IL-8 pg/mL

- **Residuals:** Indicates the spread of residuals (errors). Large values suggest potential outliers or a non-linear relationship.
- **Coefficients:**
  - None of the predictors (Cohort, Varsta, Sex, IMC) are statistically significant (p-values > 0.05), meaning there's no evidence they affect IL-8 levels.
- **R-squared:** 0.002302, very low, indicating the model explains very little of the variability in IL-8 levels.
- **F-statistic:** 0.02307 (p-value: 0.9989), the model as a whole is not statistically significant.

ELISA IL-18 pg/mL

- **Residuals:** The spread shows variation in the IL-18 levels.
- **Coefficients:**
  - Cohortcontrol (p-value = 0.0809) and the Intercept (p-value = 0.0607) are close to being significant.
  - None of the other predictors are significant.
- **R-squared:** 0.1898, low, meaning the model explains some variability in IL-18 levels.
- **F-statistic:** 2.343 (p-value: 0.0712), the model is close to being significant at the 0.05 level.

ELISA RANTES pg/mL

- **Residuals:** Indicates high variability and potential outliers.
- **Coefficients:**
  - None of the predictors are statistically significant (p-values > 0.05).
- **R-squared:** 0.03822, very low, meaning the model explains very little of the variability in RANTES levels.
- **F-statistic:** 0.3974 (p-value: 0.8093), the model as a whole is not statistically significant.

ELISA PD-L1 pg/mL

- **Residuals:** Indicates spread in PD-L1 levels.
- **Coefficients:**
  - The Intercept is significant (p-value = 0.00648).
  - SexM is close to being significant (p-value = 0.05754).
- **R-squared:** 0.1856, low, meaning the model explains some variability in PD-L1 levels.
- **F-statistic:** 2.28 (p-value: 0.07755), the model is close to being significant.

ELISA MCP-1 pg/mL

- **Residuals:** Shows variability in MCP-1 levels.
- **Coefficients:**
  - Varsta is significant (p-value = 0.0191), indicating an effect on MCP-1 levels.
  - Other predictors are not significant.
- **R-squared:** 0.2283, low, meaning the model explains some variability in MCP-1 levels.
- **F-statistic:** 2.958 (p-value: 0.03124), the model is significant.

Glicemie (mg/dl)

- **Residuals:** Indicates spread in glucose levels.
- **Coefficients:**
  - Varsta is significant (p-value = 0.0115), indicating an effect on glucose levels.
  - Other predictors are not significant.

- **R-squared:** 0.264, low, meaning the model explains some variability in glucose levels.
- **F-statistic:** 3.587 (p-value: 0.01366), the model is significant.

Hemoglobin (g/dl) Model

- **Intercept:** The baseline level of Hemoglobin when all predictors are zero is approximately 13.71 g/dl.
- **book$Cohortcontrol:** The coefficient is -1.12, but it's not statistically significant (p = 0.413), meaning there's no strong evidence to suggest that being in the control cohort significantly affects Hemoglobin levels.
- **Varsta (Age):** The coefficient is -0.001, and it's not statistically significant (p = 0.963), indicating that Age does not have a significant impact on Hemoglobin levels.
- **SexM:** The coefficient is 1.19, but it's not statistically significant (p = 0.105), suggesting that Sex (male) does not have a strong effect on Hemoglobin levels.
- **book$IMC (kk/m2):** The coefficient is -0.0003, with a high p-value (0.993), showing that BMI does not have a significant effect on Hemoglobin.

Albumin (g/dl) Model

- **Intercept:** The baseline level of Albumin is approximately 5.00 g/dl.
- **book$Cohortcontrol:** The coefficient is 0.09, but it's not statistically significant (p = 0.7726), so being in the control cohort doesn't significantly affect Albumin levels.
- **Varsta (Age):** The coefficient is -0.008, with a p-value of 0.1198, which is not statistically significant, indicating Age might not have a significant effect on Albumin.
- **SexM:** The coefficient is 0.31, with a p-value of 0.0648, which is close to the 0.05 threshold, suggesting a possible but not definitive effect of Sex on Albumin.
- **book$IMC (kk/m2):** The coefficient is -0.0127, with a p-value of 0.1378, showing that BMI does not have a significant effect on Albumin levels.

Total Proteins (g/dl) Model

- **Intercept:** The baseline level of Total Proteins is approximately 7.24 g/dl.
- **book$Cohortcontrol:** The coefficient is 0.99 with a p-value of 0.0165, which is statistically significant. Being in the control cohort is associated with higher Total Proteins.
- **Varsta (Age):** The coefficient is -0.0105, with a p-value of 0.1027, which is not statistically significant, indicating Age might not have a significant effect on Total Proteins.
- **SexM:** The coefficient is 0.45, with a p-value of 0.0345, suggesting that being male is significantly associated with higher Total Proteins.
- **book$IMC (kk/m2):** The coefficient is 0.0036, with a p-value of 0.7301, showing that BMI does not significantly affect Total Proteins.

ALAT/TGP (U/L)

- **Intercept:** Baseline value of ALAT/TGP is approximately 90.28 U/L.
- **book$Cohortcontrol:** Coefficient of -34.88, but not statistically significant (p = 0.0710), indicating a possible but not conclusive decrease in ALAT/TGP for the control cohort.
- **Varsta (Age):** Coefficient of -0.54, not statistically significant (p = 0.1066), suggesting Age does not have a significant impact.
- **SexM:** Coefficient of 13.44, not statistically significant (p = 0.1576), implying Sex (male) does not significantly affect ALAT/TGP.
- **book$IMC (kk/m2):** Coefficient of -0.75, not statistically significant (p = 0.2879), indicating BMI does not have a significant effect.

ASAT/TGO (U/L)

- **Intercept:** Baseline value of ASAT/TGO is approximately 77.75 U/L.
- **book$Cohortcontrol:** Coefficient of -30.73, statistically significant (p = 0.03449), suggesting that being in the control cohort significantly reduces ASAT/TGO levels.

- **Varsta (Age):** Coefficient of -0.14, not statistically significant (p = 0.56146), indicating Age does not have a significant effect.
- **SexM:** Coefficient of 9.06, not statistically significant (p = 0.20054), implying Sex (male) does not significantly affect ASAT/TGO.
- **book$IMC (kk/m2):** Coefficient of -1.00, marginally significant (p = 0.05999), suggesting a possible negative effect of BMI on ASAT/TGO.

Urea (mg/dl)

- **Intercept:** Baseline value of Urea is approximately 15.72 mg/dl.
- **book$Cohortcontrol:** Coefficient of -1.73, not statistically significant (p = 0.79659), suggesting that being in the control cohort does not significantly affect Urea levels.
- **Varsta (Age):** Coefficient of 0.40, statistically significant (p = 0.00157), indicating that Age has a significant positive effect on Urea levels.
- **SexM:** Coefficient of -0.19, not statistically significant (p = 0.95546), implying Sex (male) does not significantly affect Urea.
- **book$IMC (kk/m2):** Coefficient of 0.02, not statistically significant (p = 0.92115), indicating BMI does not have a significant effect.

Creatinine (mg/dl)

- **Intercept:** Baseline value of Creatinine is approximately 0.60 mg/dl.
- **book$Cohortcontrol:** Coefficient of 0.09, not statistically significant (p = 0.333360), suggesting no significant effect from being in the control cohort.
- **Varsta (Age):** Coefficient of 0.002, not statistically significant (p = 0.208738), indicating Age does not have a significant effect.
- **SexM:** Coefficient of 0.15, statistically significant (p = 0.001612), indicating that Sex (male) significantly affects Creatinine levels.
- **book$IMC (kk/m2):** Coefficient of -0.00002, not statistically significant (p = 0.995177), showing BMI does not have a significant effect.

HDL Cholesterol (mg/dl)

- **Intercept:** Baseline value of HDL Cholesterol is approximately 48.51 mg/dl.
- **book$Cohortcontrol:** Coefficient of 16.69, statistically significant (p = 0.03511), indicating that being in the control cohort is associated with higher HDL Cholesterol.
- **Varsta (Age):** Coefficient of 0.09, not statistically significant (p = 0.48665), suggesting Age does not have a significant effect.
- **SexM:** Coefficient of -5.28, not statistically significant (p = 0.17192), indicating Sex (male) does not significantly affect HDL Cholesterol.
- **book$IMC (kk/m2):** Coefficient of -0.11, not statistically significant (p = 0.69512), showing BMI does not have a significant effect.

LDL Cholesterol (mg/dl)

- **Intercept:** Baseline value of LDL Cholesterol is approximately 205.95 mg/dl.
- **book$Cohortcontrol:** Coefficient of -35.51, marginally significant (p = 0.06348), suggesting a possible decrease in LDL Cholesterol for the control cohort.
- **Varsta (Age):** Coefficient of -0.36, not statistically significant (p = 0.27089), indicating Age does not significantly affect LDL Cholesterol.
- **SexM:** Coefficient of -4.00, not statistically significant (p = 0.66659), suggesting Sex (male) does not have a significant effect.
- **book$IMC (kk/m2):** Coefficient of -1.95, statistically significant (p = 0.00719), indicating that BMI has a significant negative effect on LDL Cholesterol levels.

Total Cholesterol (mg/dl)

- **Intercept:** Baseline value of Total Cholesterol is approximately 301.10 mg/dl.
- **book$Cohortcontrol:** Coefficient of -50.07, marginally significant ($p = 0.0522$), suggesting a possible decrease in Total Cholesterol for the control cohort.
- **Varsta (Age):** Coefficient of -0.14, not statistically significant ($p = 0.7468$), indicating Age does not have a significant effect.
- **SexM:** Coefficient of -3.18, not statistically significant ($p = 0.7991$), suggesting Sex (male) does not significantly affect Total Cholesterol.
- **book$IMC (kk/m2):** Coefficient of -2.59, statistically significant ($p = 0.0079$), indicating that BMI has a significant negative effect on Total Cholesterol levels.

Triglycerides (mg/dl)

- **Intercept:** Baseline value of Triglycerides is approximately 172.11 mg/dl.
- **book$Cohortcontrol:** Coefficient of -78.70, not statistically significant ($p = 0.0870$), suggesting a possible but not conclusive decrease in Triglycerides for the control cohort.
- **Varsta (Age):** Coefficient of 1.09, not statistically significant ($p = 0.1703$), indicating Age does not have a significant effect.
- **SexM:** Coefficient of 9.88, not statistically significant ($p = 0.6592$), implying Sex (male) does not significantly affect Triglycerides.
- **book$IMC (kk/m2):** Coefficient of -1.66, not statistically significant ($p = 0.3230$), showing BMI does not have a significant effect.

Baseline – followup

t_test_p_value: The p-value from the paired t-test.

wilcox_test_p_value: The p-value from the Wilcoxon signed-rank test.

Variable	Baseline_Median	Followup_Median	t_test_p_value	wilcox_test_p_value	<chr>
1 ALAT/TGP (U/L)	27.5	24	0.0603	0.0732	
2 ASAT/TGO (U/L)	19	22.5	0.266	0.950	
3 Acid uric (mg/dl)	6.64	6.01	0.655	1	
4 Albumi (g/dl)	4.1	3.97	0.0727	0.117	
5 CRP (mg/dl)	0.725	0.28	0.0107	0.0107	
6 Colesterol total (mg/dl)	184.	160.	0.794	0.258	
7 Creatini (mg/dl)	0.705	0.8	0.175	0.230	
8 ELISA IL-18 pg/mL	166	138.	0.361	0.203	
9 ELISA IL-8 pg/mL	5.12	4.44	0.423	0.804	
10 ELISA MCP-1 pg/mL	198.	157.	0.0406	0.0730	
11 ELISA PD-L1 pg/mL	23.4	25.9	0.175	0.330	
12 Glicemie (mg/dl)	86.5	81	0.139	0.177	
13 HDL colesterol (mg/dl)	44.5	52.5	0.00770	0.0142	
14 HbA1c (hemoglobi glicozilata) %	6.08	5.61	0.0434	0.00265	
15 Hemoglobi (g/dl)	13.8	12.8	0.0254	0.0238	
16 IMC (kk/m2)	44.3	32.7	0.000000178	0.0000305	
17 LDL colesterol (mg/dl)	106.	106.	0.660	0.889	
18 Leucocite (10^9/L)	9.30	6.54	0.000172	0.000122	
19 Limfocite (10^9/L)	2.53	2.26	0.232	0.397	
20 Monocite (10^9/L)	0.535	0.415	0.00224	0.00516	
21 Neutrofile (10^9/L)	6.09	3.60	0.000198	0.00109	
22 Proteine totale (g/dl)	7.18	7.02	0.161	0.127	
23 Sideremie (ug/dl)	83	86.5	0.978	0.666	
24 Trigliceride (mg/dl)	136	95.5	0.0533	0.0444	
25 Trombocite (10^9/L)	292	262	0.103	0.162	
26 Uree (mg/dl)	33	37.5	0.178	0.185	

Detailed Interpretation:

1. **ALAT/TGP (U/L)**
   - t-test: p = 0.0603 (Not significant)
   - Wilcoxon test: p = 0.0732 (Not significant)
   - Interpretation: No significant change from baseline to follow-up.
2. **ASAT/TGO (U/L)**
   - t-test: p = 0.266 (Not significant)
   - Wilcoxon test: p = 0.950 (Not significant)
   - Interpretation: No significant change from baseline to follow-up.
3. **Acid uric (mg/dl)**
   - t-test: p = 0.655 (Not significant)
   - Wilcoxon test: p = 1 (Not significant)
   - Interpretation: No significant change from baseline to follow-up.
4. **Albumi (g/dl)**
   - t-test: p = 0.0727 (Not significant)
   - Wilcoxon test: p = 0.117 (Not significant)
   - Interpretation: No significant change from baseline to follow-up.
5. **CRP (mg/dl)**
   - t-test: p = 0.0107 (Significant)
   - Wilcoxon test: p = 0.0107 (Significant)
   - Interpretation: Significant reduction from baseline to follow-up.
6. **Colesterol total (mg/dl)**
   - t-test: p = 0.794 (Not significant)
   - Wilcoxon test: p = 0.258 (Not significant)
   - Interpretation: No significant change from baseline to follow-up.
7. **Creatini (mg/dl)**
   - t-test: p = 0.175 (Not significant)
   - Wilcoxon test: p = 0.230 (Not significant)
   - Interpretation: No significant change from baseline to follow-up.
8. **ELISA IL-18 pg/mL**
   - t-test: p = 0.361 (Not significant)
   - Wilcoxon test: p = 0.203 (Not significant)
   - Interpretation: No significant change from baseline to follow-up.
9. **ELISA IL-8 pg/mL**
   - t-test: p = 0.423 (Not significant)
   - Wilcoxon test: p = 0.804 (Not significant)
   - Interpretation: No significant change from baseline to follow-up.
10. **ELISA MCP-1 pg/mL**
    - t-test: p = 0.0406 (Significant)
    - Wilcoxon test: p = 0.0730 (Not significant)
    - Interpretation: t-test indicates a significant reduction, Wilcoxon test does not confirm.
11. **ELISA PD-L1 pg/mL**
    - t-test: p = 0.175 (Not significant)
    - Wilcoxon test: p = 0.330 (Not significant)
    - Interpretation: No significant change from baseline to follow-up.
12. **Glicemie (mg/dl)**
    - t-test: p = 0.139 (Not significant)
    - Wilcoxon test: p = 0.177 (Not significant)
    - Interpretation: No significant change from baseline to follow-up.
13. **HDL colesterol (mg/dl)**
    - t-test: p = 0.00770 (Significant)
    - Wilcoxon test: p = 0.0142 (Significant)
    - Interpretation: Significant reduction from baseline to follow-up.
14. **HbA1c (hemoglobi glicozilata) %**
    - t-test: p = 0.0434 (Significant)
    - Wilcoxon test: p = 0.00265 (Significant)
    - Interpretation: Significant reduction from baseline to follow-up.
15. **Hemoglobi (g/dl)**
    - t-test: p = 0.0254 (Significant)

    - Wilcoxon test: p = 0.0238 (Significant)
    - Interpretation: Significant reduction from baseline to follow-up.
16. **IMC (kk/m2)**
    - t-test: p = 0.000000178 (Highly significant)
    - Wilcoxon test: p = 0.0000305 (Highly significant)
    - Interpretation: Significant reduction from baseline to follow-up.
17. **LDL colesterol (mg/dl)**
    - t-test: p = 0.660 (Not significant)
    - Wilcoxon test: p = 0.889 (Not significant)
    - Interpretation: No significant change from baseline to follow-up.
18. **Leucocite (10^9/L)**
    - t-test: p = 0.000172 (Highly significant)
    - Wilcoxon test: p = 0.000122 (Highly significant)
    - Interpretation: Significant reduction from baseline to follow-up.
19. **Limfocite (10^9/L)**
    - t-test: p = 0.232 (Not significant)
    - Wilcoxon test: p = 0.397 (Not significant)
    - Interpretation: No significant change from baseline to follow-up.
20. **Monocite (10^9/L)**
    - t-test: p = 0.00224 (Significant)
    - Wilcoxon test: p = 0.00516 (Significant)
    - Interpretation: Significant reduction from baseline to follow-up.
21. **Neutrofile (10^9/L)**
    - t-test: p = 0.000198 (Highly significant)
    - Wilcoxon test: p = 0.00109 (Highly significant)
    - Interpretation: Significant reduction from baseline to follow-up.
22. **Proteine totale (g/dl)**
    - t-test: p = 0.161 (Not significant)
    - Wilcoxon test: p = 0.127 (Not significant)
    - Interpretation: No significant change from baseline to follow-up.
23. **Sideremie (ug/dl)**
    - t-test: p = 0.978 (Not significant)
    - Wilcoxon test: p = 0.666 (Not significant)
    - Interpretation: No significant change from baseline to follow-up.
24. **Trigliceride (mg/dl)**
    - t-test: p = 0.0533 (Not significant)
    - Wilcoxon test: p = 0.0444 (Significant)
    - Interpretation: Wilcoxon test indicates a significant reduction, t-test does not confirm.
25. **Trombocite (10^9/L)**
    - t-test: p = 0.103 (Not significant)
    - Wilcoxon test: p = 0.162 (Not significant)
    - Interpretation: No significant change from baseline to follow-up.
26. **Uree (mg/dl)**
    - t-test: p = 0.178 (Not significant)
    - Wilcoxon test: p = 0.185 (Not significant)
    - Interpretation: No significant change from baseline to follow-up.

Analysis between Cytokines and days of hospitalization (figure 4)

- **IL-18**
    - **Intercept**: The starting concentration of the cytokine is estimated at 48.2, with a high standard error of 60.4, leading to a p-value of 0.432, which means it's not statistically significant.
    - **Days**: Each additional day is associated with an increase of 13.5 in cytokine concentration, but this result is also not significant (p = 0.192).
- **IL-8**
    - **Intercept**: A starting concentration of 3.74, which is not statistically significant (p = 0.780).
    - **Days**: A small and non-significant increase in concentration per day (estimate = 1.44, p = 0.517).
- **MCP-1**
    - **Intercept**: High starting concentration of 144, not significant (p = 0.107).

    - **Days**: Increase per day also not significant (p = 0.440).
- **PD-L1**
    - **Intercept**: Statistically significant starting concentration (p = 0.000000186), meaning a very strong likelihood it differs from zero.
    - **Days**: A negative change in concentration per day (-0.610), but this is not statistically significant (p = 0.371).
- **RANTES**
    - **Intercept**: Very high starting concentration of 7068, which is significant (p = 0.045).
    - **Days**: Very large standard error for the Days estimate, resulting in a non-significant increase per day (p = 0.689).
